# Cyclitis and Irido-Cyclitis—I

**Published:** 1908-07-18

**Authors:** 


### Ophthalmology.

### CYCLITIS AND IRIDO-CYCLITIS.—I.

One of the most intractable and frequently disastrous inflammations which can attack an eyeball is that termed "cyclitis" or "uveitis." The name is derived, apparently, from the similarity of the whole uveal tract, dark in colour and hanging from the optic nerve as from a stalk, to a dark grape (uvea).

The iris may or may not be involved in the inflammation; if it is, then iridocyclitis is said to be present. Cyclitis may be present without iritis, but iritis never without some cyclitis. The reasons of the severity of this inflammation, of its long continuance, and of its destructiveness as far as vision is concerned are not difficult to understand when the extent of the uveal tract is taken into consideration. Embryologically, the uveal tract forms a completely closed hollow sphere within the eyeball, consisting of the choroid posteriorly, the ciliary body and processes, iris, and ligamentum pectinatum in the middle, and Descemet's membrane lining the inner surface of the cornea anteriorly. Hence an inflammation in any part of the uveal tract may very readily spread throughout the whole of it. An inflammation in the anterior portion extends backwards to the ciliary body, while one in the posterior portion (choroiditis) may extend forwards, bringing about a like result; the natural sequence of this is that an inflammation commencing in the middle portion (ciliary body or iris or both) tends to spread both ways, to become more severe, and to cause lasting and destructive injury not only to vision, but to the eyeball itself.

Forming the middle coat of the eyeball, situated between the retina on the inside, the sclera and conjunctiva on the outside, the uveal tract forms the great blood-supplying area of the eyeball; it nourishes both the sclera and retina to some extent, though each has also a separate supply; the rest of the eyeball, however, including the lens, depends entirely upon it for nourishment. Hence any disturbance of its function by inflammation may have far-reaching results, immediate or remote; a curious plaque-like lens opacity is an example of the latter.

A severe inflammation of the ciliary body may bring about destruction of the whole globe, leaving a mere sightless stump, soft to the touch; and, since a recurrence is probable in an eye which has resisted one attack, the same result may be brought about, but at a much later date. The aqueous humour being secreted by the ciliary processes, any inflammation therein irritates them, and as a first result increases their secretion, giving rise to a slight increase in intra-ocular tension; but almost invariably after this first phase of over-activity an inhibition of secretive power occurs, and the tension becomes less than normal; eyes which have become disorganised by cyclitis, or repeated attacks of it, are usually soft. During the active inflammatory stage of cyclitis, the triangular space between the anterior capsule of the lens and the iris, known as the posterior chamber, becomes filled with cellular and fibrinous exudate, and unless the pupil is fully dilated the iris may become adherent in its whole width to the capsule of the lens; this causes seclusio pupillæ, thus completely shutting off the anterior from the vitreous chamber in which the ciliary processes are located and into which the secretion of aqueous takes place. Further the iris itself begins to throw out exudate at its free margin, and this spreads across the pupillary opening, shuts it off (occlusio pupillæ) and binds the iris down immovably to the capsule of the lens (posterior synechia), thus still more completely closing the only free outlet for the aqueous. The immediate result thereof is an increase of intra-ocular tension to such an extent as to cause glaucoma and blindness. When only the pupillary margin of the iris becomes adherent to the lens capsule, the pressure of the fluid secreted in the vitreous chamber tends to bulge the iris forwards into the anterior chamber; but being attached at its free margin, only the middle portion can actually bulge, and this appearance of an iris markedly convex towards the anterior chamber is termed "iris bombé." The secondary result of the obliteration of the posterior chamber by fibrinous exudate and its subsequent contraction, whereby the iris is firmly bound down to the anterior capsule of the lens, is a deepening of the anterior chamber due to the retraction of the iris, one of the classic clinical signs of iridocyclitis.

The rise in intra-ocular tension during the attack of cyclitis is one of the most difficult phases in this inflammation to deal with effectively. If atropine be withheld the posterior chamber will most certainly become occluded; on the other hand, to continue with it seems like courting certain disaster in the shape of an attack of acute glaucoma. What frequently saves the situation is the fact that the filtration angle between the root of the iris and the posterior surface of the cornea remains open, for a time at least, and this enables the anterior chamber to free itself; also that the ciliary processes, after passing through the initial irritative stage with excessive secretion, begin to secrete even less than normal, thus helping still further to keep the tension within bounds. Some circulation of fluid may take place from the posterior chamber to the canal of Schlemm and spaces of Fontana through the root of the iris, unless this has been blocked by excessive secretion or very turbid aqueous. If, however, the tension of the eyeball remains too high, causing great pain and minimising the chance of the vision remaining useful after the attack, the anterior chamber should be evacuated, if necessary several times.

Finally the inflammation begins to subside, with lessened congestion of the vessels within the eyeball, the tension becomes more nearly normal, the eyeball becomes again white, the pain—both the dull ache and the neuralgic species round the orbit—ceases, and finally the eye settles down, sometimes only slightly damaged, but at others unfortunately ruined as an organ of vision.

